# Towards resilience through systems-based plant breeding. A review

**DOI:** 10.1007/s13593-018-0522-6

**Published:** 2018-08-22

**Authors:** Edith T. Lammerts van Bueren, Paul C. Struik, Nick van Eekeren, Edwin Nuijten

**Affiliations:** 1 Louis Bolk Institute, Kosterijland 3-5, 3981 AJ Bunnik, The Netherlands; 2 Department of Plant Sciences, Wageningen UR Plant Breeding, Wageningen University and Research, P.O. Box 386, 6700 AJ Wageningen, The Netherlands; 3 Department of Plant Sciences, Centre for Crop Systems Analysis, Wageningen University and Research, P.O. Box 430, 6700 AK Wageningen, The Netherlands

**Keywords:** Agrobiodiversity, Breeding strategies, Common good, Ecological resilience, Entrepreneurial models, Resource use efficiency, Seed systems, Social justice, Societal resilience, Sustainability

## Abstract

How the growing world population can feed itself is a crucial, multi-dimensional problem that goes beyond sustainable development. Crop production will be affected by many changes in its climatic, agronomic, economic, and societal contexts. Therefore, breeders are challenged to produce cultivars that strengthen both ecological and societal resilience by striving for six international sustainability targets: food security, safety and quality; food and seed sovereignty; social justice; agrobiodiversity; ecosystem services; and climate robustness. Against this background, we review the state of the art in plant breeding by distinguishing four paradigmatic orientations that currently co-exist: community-based breeding, ecosystem-based breeding, trait-based breeding, and corporate-based breeding, analyzing differences among these orientations. Our main findings are: (1) all four orientations have significant value but none alone will achieve all six sustainability targets; (2) therefore, an overarching approach is needed: “systems-based breeding,” an orientation with the potential to synergize the strengths of the ways of thinking in the current paradigmatic orientations; (3) achieving that requires specific knowledge development and integration, a multitude of suitable breeding strategies and tools, and entrepreneurship, but also a change in attitude based on corporate responsibility, circular economy and true-cost accounting, and fair and green policies. We conclude that systems-based breeding can create strong interactions between all system components. While seeds are part of the common good and the basis of agrobiodiversity, a diversity in breeding approaches, based on different entrepreneurial approaches, can also be considered part of the required agrobiodiversity. To enable systems-based breeding to play a major role in creating sustainable agriculture, a shared sense of urgency is needed to realize the required changes in breeding approaches, institutions, regulations and protocols. Based on this concept of systems-based breeding, there are opportunities for breeders to play an active role in the development of an ecologically and societally resilient, sustainable agriculture.

## Contents

- 1. Introduction
- 2. The roles and positioning of plant breeding and seed systems in food systems
- 3. Diverse orientations in plant breeding and the seed sector
- 3.1. Community-based breeding
- 3.2. Ecosystem-based breeding
- 3.3. Trait-based breeding
- 3.4. Corporate-based breeding
- 3.5. The need to combine potentials of the four paradigmatic orientations
- 4. Defining a systems-based breeding orientation
- 4.1. Required change in attitude
- 4.2. From attitude to action
- 4.3. From action to achievement
- 5. Discussion and conclusions
- 5.1. Diversity of breeding initiatives and variety registration protocols
- 5.2. Seeds as common good
- 5.3. New entrepreneurial models
- 5.4. Shared sense of urgency
- 5.5. Perspective
- References

## Introduction

Since the 1950s, agricultural policies were very conducive to increasing yield per hectare and per unit of labor. Consequently, plant breeding was strongly oriented towards creating cultivars that were highly productive and suitable for machine-harvesting in large-scale, high-external-input farming systems (Fraser et al. 2016; Bradshaw 2017). However, during the last few decades, awareness of the negative side-effects of these policies, for example on the environment, the sustainability of food production systems, the agrobiodiversity and ecosystem services of agroecosystems, has been increasing. It became apparent that a societal debate is required to assess and evaluate multi-dimensional trade-offs in food production, with respect for and accepting differences in norms and values (Struik et al. 2014; Struik and Kuyper 2017). We need to rethink and reorganize our food systems, i.e., the way we produce, harvest, store, transport, process, market, and consume (or dispose of) our food.

In addition to the need to restore the sustainability of the production systems, food security, food safety, food quality, and food sovereignty for a rapidly growing and increasingly demanding world population are urgent issues requiring continuous crop improvement, yield gains, and reductions of losses of produce during harvesting, storage, and processing (FAO 2016; FAO et al. 2017). Most scientists assume that realizing these demands requires doubling global food production by 2050 (for a debate on this need, see Tomlinson 2013), based on sustainable intensification. FAO (2001) defined food security as “a situation that exists when all people, at all times, have physical, social and economic access to sufficient, safe and nutritious food that meets their dietary needs and food preferences for an active and healthy life.” Food safety is part of that food security concept: it is the absence of harmful or health-threatening components in the food. Positive aspects related to nutritious and healthy food need to be addressed more explicitly (Dwivedi et al. 2017). In this paper, it is covered in the phrase food quality. Food sovereignty is a much wider concept than food security: it is the right of local communities to define and control their own food systems (see, e.g., Wittman 2011). Food sovereignty encompasses the equity and ecological foundations for the achievement of food security, including farmer autonomy and sustainable use of natural resources, actively involving urban dwellers (especially women) in the food system, and not merely as consumers, and free access to and control over seed (Bezner Kerr 2010; Snapp et al. 2010; Wittman 2011; Alkon 2013; Barthel et al. 2013; Montenegro de Wit 2016). Food sovereignty includes self-organized systems of rules and diverse packages of practices and technologies from creating varieties, producing seed, growing crops, processing the harvest, all the way to marketing the produce (Barthel et al. 2013). The diversity of crops and food sources is an issue in itself as it affects the stability of the global food system. There is concern that crop diversity is in decline (Khoury et al. 2014). Here, we stress that food sovereignty is only possible when also seed sovereignty is guaranteed, i.e., when agrobiodiversity and the resulting seed (system) are considered as commons and public good and managed based on the rules that apply for common-pool resources (Ostrom 2008).

Biodiversity and ecosystem services are key factors that regulate and support the environment within agro-ecosystems, sustain future food production, and contribute to natural pest control, pollination, nutrient (re)cycling, soil conservation (structure and fertility), water provision (quality and quantity), carbon sequestration, etc. (Power 2010; Harrison et al. 2014; Huang et al. 2015), i.e., they contribute to sustainable intensification. FAO (2011) defined sustainable intensification as “producing more from the same area of land while conserving resources, reducing negative impacts on the environment and enhancing natural capital and the flow of ecosystem services,” although the entire concept of sustainable intensification has been disputed (Struik et al. 2014; Struik et al. 2017). It is only possible with substantial and long-term efforts, not only from agronomists and breeders, but also from other players in the food system, such as processors, retailers, and consumers. At the same time, agricultural production must also be made climate-robust, i.e., all players in the food system, including plant breeders, must find ways to mitigate the negative effects of climate change, with its increasingly unpredictable and extreme weather patterns, on the food system. Obviously, increasing yields should not go to the detriment of nutritional quality, taste and other food qualities.

With such enormous tasks ahead, one would expect that farmers, breeders, scientists, and other involved chain actors would get all the support from national and international governments and institutions required to achieve their goals. Unfortunately, this is not the case: agriculture, breeding, and agronomy have become contested despite their great successes (Khush 2001) and they are currently confronted with multifaceted challenges set by national and supra-national policy targets, environmentalists, and pressure groups (Kiers et al. 2008; Brussaard et al. 2010; Power 2010; Lauer et al. 2012; Sumberg et al. 2013). These challenges cannot be met by technical solutions alone, but require solutions that take socio-economic, ethical, and judicial aspects into account, such as social justice (for example equal access to knowledge and technologies) (Tilman et al. 2002; Kiers et al. 2008; Brussaard et al. 2010; Koohafkan et al. 2012; Kuyper and Struik 2014; Struik and Kuyper 2014; Struik et al. 2014; United Nations 2015; FAO 2016; FAO et al. 2017; Struik and Kuyper 2017). Therefore, an integrated approach is needed based on comparative and quantitative analyses of trade-offs (with multiple temporal and spatial dimensions) to strengthen ecological resilience in combination with societal debates on norms and values, negotiations between stakeholders, and subsequent political choices on how to produce (Hisschemöller and Hoppe 1995; Kiers et al. 2008; Struik and Kuyper 2017) in order to strengthen societal resilience.

Starting in the 1980s, various scientists have substantiated that, in addition to breeders, farmers and other chain actors have a role to play in improving ecological resilience through selecting for higher yielding varieties (Sperling et al. 1993; Ceccarelli et al. 2001) and maintaining high levels of crop diversity (Teshome et al. 1997; Zimmerer 1998). From the 1990s onwards, initiatives were developed to improve societal resilience through so-called participatory plant breeding (Sperling et al. 2001), in some cases with a particular focus on the empowerment of women (Galiè et al. 2017). However, valuable lessons learned from these studies have not reached the mainstream debate on the future of agriculture.

In short, the necessary sustainable intensification of agriculture aiming at both ecological and societal resilience requires a major effort from politics, society, farmers, other value chain actors, and science. In this context, practitioners and scientists involved in plant breeding and seed systems have a major role to play.

**Fig. 1:**
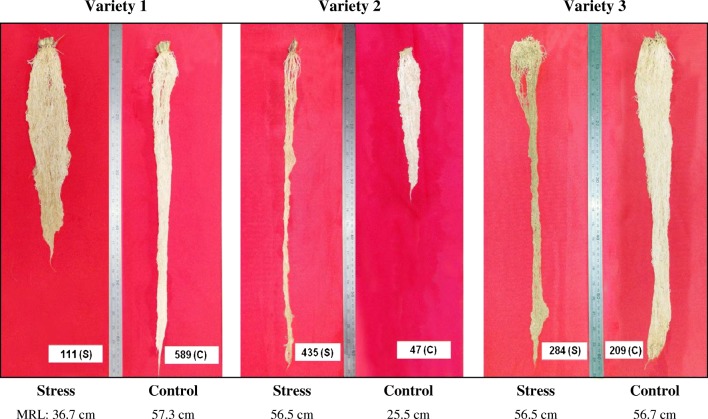
Ecological resilience in rice. The figure shows different types of phenotypic plasticity in response to water-deficit stress during the vegetative stage. Variety 1 shows a root system with a short root length under water deficit compared to the control, Variety 2 shows a longer root system under water deficit compared to the control, whereas Variety 3 shows equally long root systems for the water-deficit treatment and the control. Material and data from an experiment described by Kadam et al. (2017). Reproduced with kind permission from Dr. Niteen N. Kadam, International Rice Research Institute and Wageningen University & Research

**Fig. 2:**
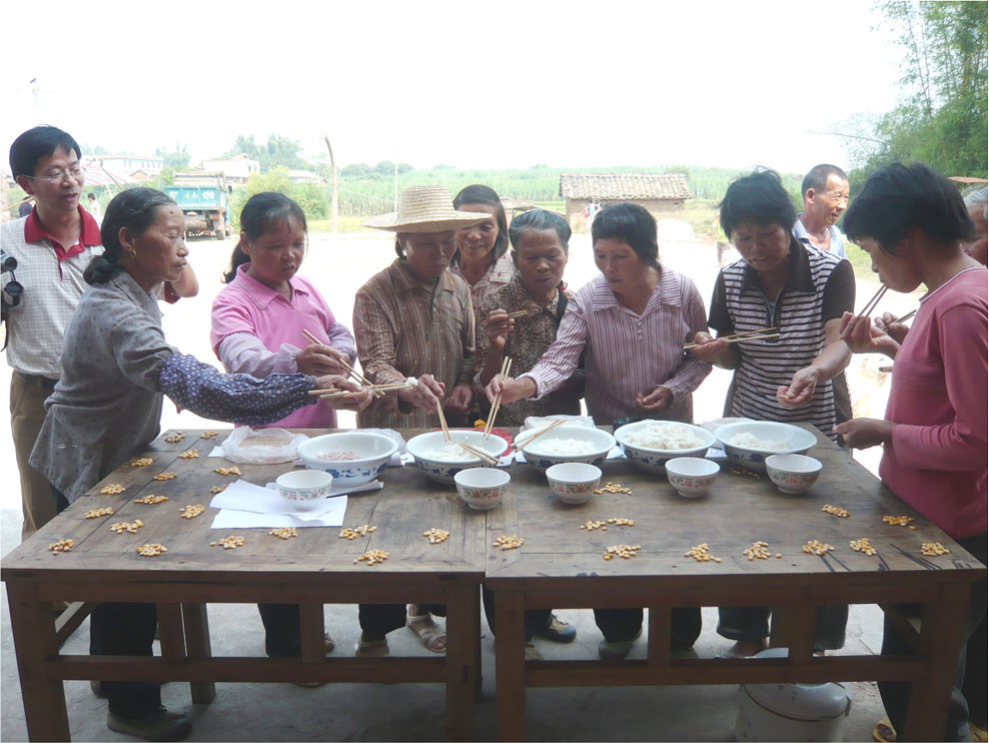
Societal resilience of rice. Chinese female farmers rank the quality of rice prepared from different cultivars based on their culturally determined preferences and allocating a certain number of maize kernels to indicate the rank of preference. Rice cultivars play an important role in local food and seed sovereignty. Picture by Edith T. Lammerts van Bueren

Figures 1 and 2 provide some examples of ecological and societal resilience.

In this paper, we analyze and discuss the role of plant breeding and seed systems in designing the future directions towards meeting the six international policy targets on agro-biodiversity; climate robustness; ecosystem services; food security, safety and quality; food and seed sovereignty; and social justice. In order to evaluate (i) how plant breeding has shaped the development of agricultural production, including both positive and unintended negative side-effects, (ii) how it can contribute to mitigating these negative side-effects, (iii) how it can become involved in the debate and negotiations on how to re-organize our food systems, and (iv) how it can contribute to meeting current and future challenges, we will first describe the position of the breeding and seed systems within their economic, institutional and cultural context. We will then analyze four existing orientations of plant breeding, and define their main characteristics, strengths and weaknesses. We will use that analysis to propose a new concept, which we coin “systems-based breeding” and which will help plant breeders to navigate when they are designing new programs to support the creation of sustainable food systems. Finally, we will also discuss key trends that need to be supported on the route towards such systems-based breeding.

## The roles and positioning of plant breeding and seed systems in food systems

**Fig. 3:**
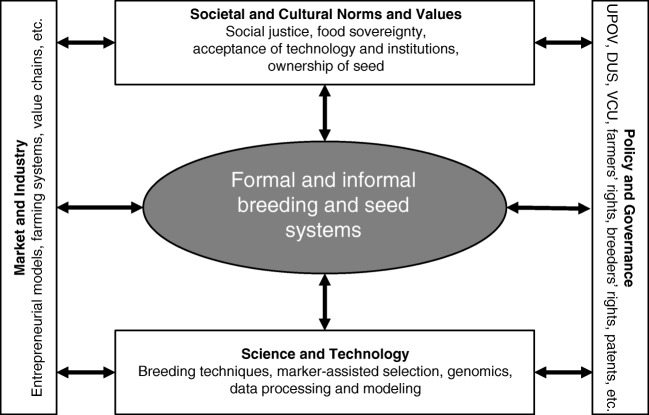
Roles and positioning of the breeding and seed systems within their technical, economic, institutional and cultural context

Breeding and seed systems have specific roles and are influenced by a complexity of socio-economic, historical, and socio-political factors. An illustration of these roles and influences is necessary to understand the playing field in which the breeding and seed systems are positioned and in which choices can be made that will direct future opportunities. Figure 3 illustrates the roles, positioning, and interwovenness of formal plant breeding and seed systems (i.e., described by official law and regulations, leading to certified seed of verified varieties) and informal plant breeding and seed systems (farmer-led, including farmers’ variety selection, seed production, and seed exchange activities), in food systems with various types of societal factors. These factors include: (i) the markets and their value chains, (ii) policy and governance in relation to genetic resources, variety testing and registration, intellectual property rights, etc., (iii) science and technology supporting the development of breeding products and tools, and (iv) societal and cultural norms and values defining acceptability of approaches and products.

During the past century, the nature of breeding in industrialized countries has changed substantially from farm-based seed production and selection activities leading to locally adapted landraces via commercial breeding supported by publicly funded breeding of genitors (e.g., with new resistance genes from wild relatives), more and more towards a science- and corporate-based activity concentrated in highly specialized and internationally operating multinationals. Parallel to these changes in breeding systems, the seed systems in industrialized countries developed from open, informal seed systems based on farm-saved seed and community seed networks based on sharing and exchange, supporting seed sovereignty, towards formal, more closed seed systems with strong oversight and certification systems to guarantee genetic, physical, physiological, and phytosanitary quality of seed. In these formal seed systems, the seed multiplication became a corporate specialization, where farmers faced dispossession and lost sovereignty over seed; farmers lost the opportunity to multiply their own seed on-farm, either for biological reasons (through the provision of irreproducible F1 hybrid seeds) or for legal reasons (based on restrictive seed laws on farm-saved seeds or by patent rules) (Kloppenburg 2010). These developments denied the positive role of farmers or community seed networks in maintaining agricultural diversity (Coomes et al. 2015 and references therein).

Simultaneously, public plant breeding and variety testing schemes have been strongly down-sized, while private companies concentrate more and more on a limited assortment of cash crops in an economically highly competitive market. Currently, mainstream large plant breeding companies in industrialized countries have their own Research and Development departments to increase speed and efficiency of the breeding process including marker-assisted selection based on the opportunities offered by genomics (and other “omics”) in combination with advanced processing techniques of big data and modeling approaches (Yin and Struik 2016). These developments require large investments in technologies and human resources. The rapid increase in technological opportunities in the primary process of creating genetic variation and breeding material that enabled the use of this genetic variation and the testing of new breeding material increased the costs of bringing new cultivars to the market and changed the playing field for breeders drastically in a very short time.

These developments had several disadvantages or negative side-effects. For example, many useful crops with a relatively small acreage became orphans for which breeding is no longer carried out (Gepts and Hancock 2006; Khoury et al. 2014; Bradshaw 2017), thus increasing the dependence of the world food system on a limited number of crops (Khoury et al. 2014). Moreover, the policy and governance rules involved in variety testing protocols, variety registration, and on-farm seed saving, once developed to protect the seed users (farmers), now more and more seem to develop into institutions that protect the interests of the breeding industry (Louwaars et al. 2011; Braunschweig et al. 2014).

These developments triggered resistance from societal organizations: breeding has become contested, for example for reasons of social justice, food sovereignty, ownership of genetic resources, farmers’ rights on seed saving (seed sovereignty), patenting of plant material, climate-robust traits and genes, improvement of small crops to maintain crop diversity, acceptance of novel breeding techniques, the mergers and monopolies of large multinationals, and the package deals of genetic modification (GM) technology associated with the use of pesticide resistant cultivars (Madsen and Sandøe 2005; Halewood et al. 2007; Waltz 2009; Jacobsen et al. 2013; Ceccarelli 2014; De Schutter 2014; Lucht 2015; Nuijten et al. 2017a).

Seeds are an essential element, not only of our food systems but also of our culture and society as a whole. Therefore, plant breeding should be in the center of the societal debate on the future of agriculture. However, a debate on the role of plant breeding in an integrated approach to meet the policy targets on ecological and societal resilience of agriculture as a whole is noticeably lacking. In the next section, we analyze the different positions (or “orientations”) in the breeding and seed sector and argue that there is a need for change to enhance the contributions of plant breeding towards ecological and societal resilience.

## Diverse orientations in plant breeding and the seed sector

**Fig. 4:**
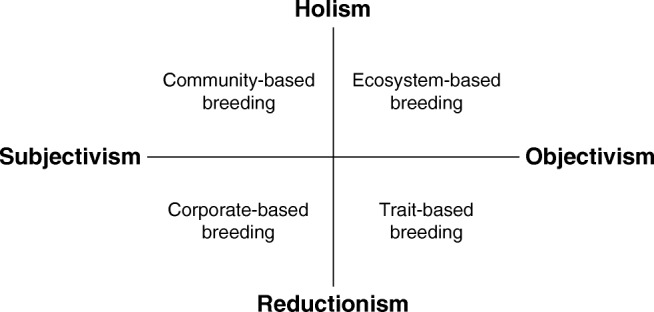
Four breeding orientations as functions of different positions between subjectivism and objectivism, and between holism and reductionism

To define the different orientations of plant breeding, we developed an analytical framework based on Bawden’s framework of worldviews (Bawden 2010). Figure 4 illustrates four breeding orientations originating from different combinations of subjectivism and objectivism on the one hand, and of holism and reductionism on the other hand. These four combinations, termed “paradigmatic positions,” have different styles of thought as visualized in the four quadrants of Fig. 4 and are called: (1) community-based breeding, (2) ecosystem-based breeding, (3) trait-based breeding, and (4) corporate-based breeding. Community-based breeding can be considered as a search for restoring or renewing alliances as part of local, innovative food systems supporting food sovereignty and cultural diversity. Ecosystem-based breeding supports developing varieties adapted to various pedo-climatic growing conditions at regional level. Corporate-based and trait-based breeding orientations constitute the currently dominant, reductionist style of thought in most industrialized countries and are represented by their commercial breeding multinationals and breeding research institutes. Corporate-based breeding aims to meet particular wishes and needs of the market, whereas trait-based breeding departs from the notion what kind of crops society needs to boost future crop production, and aims to dig deeper into the genetics behind the underlying traits. Whereas the two paradigmatic positions on the left of Fig. 4 are driven by subjective goals of commercial companies (corporate-based breeding) or communities (community-based breeding), the two paradigmatic positions on the right of Fig. 4 are driven by objective goals that support better performance of crops by disentangling traits (trait-based breeding) or by matching cultivars to the right environments (ecosystem-based breeding).

Below we will describe the four paradigmatic positions in more detail. To support these descriptions, we have added Table 1, which provides an overview of our perception of the specific characteristics of each breeding orientation with respect to the institutional philosophy and related norms and values, socio-economic aspects, breeding technology, legal aspects, and risk management. Table 2 summarizes the strengths and potential weaknesses of each breeding orientation.

**Table 1:** Overview of key characteristics of four breeding orientations

Orientation	Characteristic	Community-based	Ecosystem-based	Trait-based	Corporate-based
Institutional philosophy	Worldview	Holocentric	Ecocentric	Technocentric	Egocentric
	Style of thought	• Respecting cultural and ecological diversity • Focus on solidarity • Aiming at food and seed sovereignty and at social justice	• Focus on varieties that fit particular ecosystems aiming at improved predictability looking for general patterns • Utilizing functional diversity	• Focus on technical innovations and regulations • Aiming at improved efficiency in breeding • Looking for mechanisms at detailed level	• Focus on business and market development • Aiming at corporate profit, efficiency and market competitiveness • Requiring free market policies and good intellectual property protection
	Knowledge required	• Integration of natural and social sciences, transdisciplinary • Aiming at understanding relationship between society and breeding • Requiring participatory, action research methods; room for unpredicted spontaneous solutions (serendipity)	• Interdisciplinary, ecological, scientific knowledge • Aiming at traits that support ecosystem services; multi- purpose cultivars • Requiring understanding of non-linear interrelationships and trade-offs	• Specialized, technical science oriented knowledge • Aiming at efficient, (mostly molecular) selection tools to generate in-depth knowledge • Requiring complex algorithms	• Specialized, strategic and practical knowledge • Knowledge is power • Aiming at breeding by design in context of the market requiring knowledge on handling big data, modeling and statistical tools, as well as on marketing and bio-informatics
Socio-economic aspects	Organization of breeding	Local, farmer-led, collaborative, multi-actor approach	Can be centralized, regional; with generalists; can include scientist-led participatory approaches	Centralized; with specialists; top-down	Centralized; with specialists; goal driven and top down
	Type of economy	Local economy serving local community benefits and appreciation (food and seed sovereignty)	Circular economy to reduce externalities for long-term sustainability and ecological resilience	Linear economy, specialisms, ability to engineer plants, predictability driven	Linear economy based on maximizing input-output balances; economies of scale drive mergers to guarantee economic growth
Breeding technology	Breeding strategy in terms of adaptation	Adaptation to local ecology, culture and market; preferably with durable, quantitative stress resistance	Ecologically and climate robust and regionally adapted; preferably with durable, quantitative stress resistance	Wide adaptation to specific stresses, e.g., “climate-ready genes”; major genes; major QTLs, qualitative resistances	Adapted to anonymous markets; looking for small G × E; qualitative resistance
	Main selection environment	Field, on-farm, market, community	Field/agro-ecosystem	Laboratory, field	Field, laboratory, computer, market
	Cultivar type	Open-pollinated cultivars, modern landraces, heterogeneous population breeding (composite cross populations); concern for cultural and genetic diversity, and agrobiodiversity	Both F1-hybrids and open-pollinated cultivars; research strives for robust/flexible cultivars with improved below-ground traits to enhance resilience, such as strong interactions with beneficial soil microorganisms and improved root characteristics; synthetic varieties	F1-hybrids and pure lines, well-defined cultivars; F1-hybrids based on (cytoplasmic) male sterility, genetic modification, New Breeding Techniques; managing diversity in genes over time (resistance management)	F1-hybrids and pure lines, well-defined varieties; F1-hybrids based on (cytoplasmic) male sterility, genetic modification and New Breeding Techniques; no concern about possible decrease in crop or genetic diversity
Legal aspects and risk management	Intellectual property protection	Biological open-source approach, sharing; transparency; protecting farmers’ rights; respecting common pool resources; respecting seed and food sovereignty	Breeders’ rights with breeders’ exemption for open access to genetic resources to keep genetic base as broad as possible	Focus on breeders’ rights and patents; focus on good descriptions, definitions in contracts	Excluding, closed knowledge system: strong urge to protect investments with technologies and patents; reducing farmers’ rights to farm-saved seeds
	Risk management	Risk spreading and sharing by communities by partnering and by diversification in technologies (e.g., crop diversity and within-crop diversity); appreciating diversity of solutions	Reducing risks and trade-offs by enhancing ecological robustness and buffering capacity of cultivars; searching for intrinsic solutions	Reducing risks by optimizing efficiency and speed in breeding, and relying on various extrinsic solutions (e.g., clear regulations and chemical crop protectants) in case of trade-offs	Reducing risks by continuously putting new cultivars on the market in combination with use of chemical crop protectants, and avoiding any risks and claims by clear contracts in relation to royalties
	Use of crop protection	Refraining as much as possible from chemical crop protection as health of soil, plant, animal, human and planet are indivisible	Minimize chemical use to protect ecosystem resilience and search for solutions that can reduce production risks	Utilizing chemical crop protection when innovations come short to reduce risk and increase production efficiency	Including chemical crop protection to boost production; joint ventures between breeding and chemical industry

**Table 2:** Strengths and potential weaknesses of the four breeding orientations

	Community-based breeding	Ecosystem-based breeding	Trait-based breeding	Corporate-based breeding
Strengths	• Integrative approach • Solidarity • Focus on collaboration • Respecting cultural values	• Ecological-systematic approach • Long-term perspective • Generalists • Serving ecosystem services	• Analytical-systematic approach • Detailed in-depth knowledge • Specialists	• Entrepreneurial • Competitive • Expertise in value chains • Clear business model • Ability to make large steps forward
Potential weaknesses	• Too small scale to ensure continuity • Inward focus • Too much focus on all complex relationships, not being able to move forward • Conservative and afraid of (socio-technical) innovation • Difficult to scale up/out	• Difficult to connect to currently dominant business model • Complex • Long-term profit orientation • Too broad and forgetting in-depth analysis • Forgetting the outliers • Forgetting the people	• Not always in connection with pluriformity in society • Too much focus on details and molecular oriented • Costly and thus dependent on patenting for return on investments • Dependent on industry for investments	• Too much driven by short term profit and the market • Mergers at the cost of diversity of players and crops • Path dependencies leading to monopoly • Lack of transparency and solidarity

### Community-based breeding

In the upper left quadrant of Fig. 4, the community-based breeding orientation is a style of thought that combines holism and subjectivism. It aims to develop collaborative breeding networks and organizations involving a range of chain players at local or regional level, thereby respecting diverse cultural values and societal pluriformity (see Fig. 4 and Table 1). Hence, this breeding orientation serves both cultural diversity and agro-biodiversity, maintaining and developing a diversity of (orphan) food crops and varieties, but also emphasizing food and seed sovereignty of these communities and seed as common good (Kloppenburg 2010; Wirz et al. 2017). In developing countries, it represents breeding by local communities anchored in local economies, in many cases supported by regional NGOs and research institutes, such as the MASIPAG (Farmer-Scientist Partnership for Development) initiative in the Philippines (Bachmann 2010) and various *Campesino a Campesino* initiatives in Latin America, such as the National Association of Small Farmers (ANAP) in Cuba (Rosset et al. 2011); and it often results in empowerment of local farmer communities and sometimes more specifically women farmers (see, e.g., Almekinders 2011; Li et al. 2014; Galiè et al. 2017). In the context of the industrialized world, this breeding orientation uses multi-actor approaches, involving farmers, processors, traders, restaurant chefs, and consumers. There is an increasing number of examples of such multi-actor approaches organized or supported not only by NGOs in industrialized countries enhancing breeding for regional varieties adapted to local circular economies, such as the Organic Seed Alliance in the USA (www.seedalliance.org), Rete Semi Rurali in Italy (Campanelli et al. 2015), and Réseau Semences Paysannes in France (Desclaux et al. 2008), but also supported by publicly funded research projects at research institutes and universities, such as NOVIC in the USA and Canada (Shelton and Tracy 2015), and SOLIBAM (www.solibam.eu), DIVERSIFOOD (www.diversifood.eu) and LIVESEED (www.liveseed.eu) in the EU. This “holocentric” orientation requires development of appropriate methods of breeding, experimental designs, and variety selection that fit the local, multi-faceted complexity of the social and physical environment and create sustainable and tailor-made solutions for local actors. Actors strive for ways to realize seed sovereignty involving seed types that farmers can easily reproduce or improve (e.g., open-pollinated cultivars, landraces, and heterogeneous populations) and advocate farmers’ rights for use of farm-saved seed (see, e.g., Fitzgerald 1993; Kloppenburg 2010).

To create more buffering capacity and yield stability in low-input farming systems, the concept of heterogeneous populations, also called composite cross populations originating from evolutionary breeding, is currently further developed by various breeders active in the organic sector (Murphy et al. 2005; Ceccarelli et al. 2010; Döring et al. 2011; Murphy et al. 2016; Brumlop et al. 2017; Raggi et al. 2017). These types of populations are characterized by a high level of genetic diversity, as they consist of a mixture of many different genotypes, often with complementing below- and above-ground traits.

The phenotypes and genotypes of these diverse populations are very heterogeneous and can evolve over time under the selection pressure of the environment (evolutionary breeding), as they will adjust to changing growing conditions (as under climate change) when farmers continue to save seeds on-farm. Although less diverse, farmer-developed open-pollinated cultivars and landraces also have the potential to adapt to changing growing conditions. Such diverse reproductive material does not comply with the commonly applied criteria for variety registration based on homogeneous varieties, and can therefore not be marketed in Europe without adaptation of the variety registration protocols. The European Commission is currently experimenting by providing derogations to test and market heterogeneous populations of cereals (wheat, barley, oats, and maize) in six EU countries (Cuoco 2017).

The strengths of this orientation include the attitude of seeking solidarity and collaborative and integrative breeding approaches (Table 2). As the approach includes a transparent, bottom-up approach, the trade-offs and risks are known and shared among the community members. Moreover, farmers and other value chain actors feel empowered by community-based approaches. A weakness can be that the activities are too small-scaled and that upscaling is difficult, partly due to their specific local embeddedness and partly due to political and institutional constraints. There are opportunities to link formal and informal breeding approaches when policies start to support the development of community-based, collaborative breeding organization structures as is aimed for in the various above described projects.

### Ecosystem-based breeding

The ecosystem-based breeding orientation (in the upper right quadrant) starts from an ecological perspective and is a style of thought that combines holism and objectivism analyzing general patterns in ecology and aiming at developing varieties adapted to ecological conditions at regional level (see Fig. 4 and Table 1). In the late 1980s, participatory plant breeding started to develop in response to the Green Revolution where between the 1960 to 1980s technology packages (new cultivars responding to high external inputs) were introduced, overlooking the ecological risks of high-input farming and ignoring the needs of subsistence farmers in many remote and harsh environments. There was a need for better matching cultivars with local pedo-climatic and socio-cultural conditions and therefore enhancing yield stability and food security, and empowering farmers (e.g., Bänziger and Cooper 2001; Ceccarelli et al. 2001; Almekinders and Hardon 2006; Galiè et al. 2017).

Currently, this orientation more and more departs from the notion that there is a need to enhance productivity without harming the environment and taking care of sustainability of the agroecosystems (Rockström et al. 2009; Struik and Kuyper 2014). The acknowledgment of potential ecological risks leads to the need to manage risks through both external inputs and intrinsic solutions, such as enhancing ecological robustness and buffering capacity of cultivars (Murphy et al. 2014). Relatively recent breeding research is being developed to also include long-term goals, such as contributions to the maintenance or restoration of ecosystem services as targets in breeding programs. For example, breeding will deliver cultivars that are attractive for pollinators (Suso et al. 2016), contribute to accumulation of soil organic matter (De Deyn et al. 2008; Deru et al. 2014), enhance the biodiversity in the soil (Perez et al. 2017), or increase the nutrient use efficiency at the ecosystem level (Gilbert 2016; Lammerts van Bueren and Struik 2017).

This “ecocentric” orientation puts durable sustainability and ecological resilience up front taking into account the complexity of environmental factors influencing crop growth and ecosystem functioning. This orientation can benefit from knowledge on ecological relationships, for example the interactions of crop plants with beneficial soil organisms, such as mycorrhizas, to enhance efficient nutrient uptake (Gewin 2010; Galván et al. 2011), from knowledge on physiological processes affecting traits contributing to nitrogen use efficiency (Lammerts van Bueren and Struik 2017), and from knowledge on plant plasticity under climate change leading to irregular weather patterns (Nicotra et al. 2010). Typically, breeding initiatives in this orientation are funded publicly or through international institutions.

The strengths of this orientation are the long-term holistic perspective and the focus on ecosystem health, and sustainability and on balancing trade-offs (Table 2). However, this breeding approach includes complex traits and requires many years of experience to acquire in-depth and site-specific knowledge and innovative tools for effective selection. Breeding technologies used include field-level observations and selection activities. The weakness of this orientation is that cultural and socio-economic specificities can easily be overlooked; participatory approaches are considered by the formal breeding sector for niches, such as for organic agriculture or subsistence farmers in developing countries. For instance, breeding climate-robust cultivars is not just a technical quest, but also the local, ecological, and socio-cultural context should be integrated to obtain cultivars that are regionally adapted and will be adopted.

Another concern is that in the current breeding systems, short-term return of investments is dominating and usually business cases for long-term sustainability goals (such as breeding for ecosystem services) do not yet exist. This requires incorporating true-cost accounting on the production and impact of cultivars promising high yield but at the cost of natural resources. Investments in research on complex traits serving long-term sustainability should deserve higher priority for financing by green policies.

### Trait-based breeding

The trait-based breeding orientation is a style of thought that combines objectivism with reductionism, resulting in a mechanistic view of how plants grow (see Fig. 4 and Table 1). Louwaars (2018) argues that the term “trait breeding” becomes increasingly valid because the detailed knowledge of the genome allows high levels of precision, a close relation between genotype and phenotype, and minimal linkage drag. Crop physiology and genomics support such an approach to better understand how plants grow and how to improve them, by subdividing complex traits into smaller, manageable (heritable) components and by better understanding how these components contribute to plant traits, such as salt or drought tolerance, and how they are regulated (see, e.g., Yin et al. 2004; Munns and Richards 2007). Nowadays, efforts have been made to improve the efficiency of field-based selection with increased controllability and predictability, such as with the design of early generation variety trials with correlated data and high-throughput phenotyping methods (e.g., Cullis et al. 2006; Deery et al. 2016).

This more “technocentric” orientation departs from the notion that plants are composed of genes that can be switched off (or downregulated) in case of undesired traits or switched on (or upregulated) in case of desired traits (Cardi and Stewart 2016; Mahfouz et al. 2016). Today, trait-based breeding is associated with molecular techniques and high-throughput, high-tech phenotyping systems that collect massive data sets that are used for genome-wide association studies, often supported by novel approaches of big data analysis, advanced statistical approaches and modeling exercises. Favorable traits can also be included or reorganized by design, for example through genetic engineering techniques (Koornneef and Stam 2001; Cooper et al. 2014; Haverkort et al. 2016; Bradshaw 2017). Very specialized sciences, such as molecular biotechnology, molecular genetics, and various -omics disciplines, play an important role to support the breeding-by-design approach (Peleman and Rouppe van der Voort 2003). Much of this modern technology-driven breeding research is conducted at both universities and multinationals with public and corporate funding.

The strengths of this orientation are the creation of new, in-depth knowledge of traits, their regulation, and the relationships between genetics and crop physiology (Table 2). But this breeding orientation with its emphasis on technical solutions for complex questions can easily lead to blind spots: knowledge development is very much focused on the commercial breeding industry for large-scale farming systems and less attention is paid to other breeding models that can be applied by small-scale breeders or farmer communities, or to developing strategies for more diversified food systems (see, e.g., Lammerts van Bueren et al. 2010; Ceccarelli 2014; Dwivedi et al. 2017). Simultaneously, this approach has difficulties to relate the technical knowledge with the broader agro-ecological and socio-economic context. Similar to ecosystem-based breeding, trait-based breeding has the risk to focus merely on technical solutions and to be less oriented on specific cultural aspects, norms and values. Another weakness is the fact that capital investments in technology developed by companies or research institutes are high and that patents play an important role, not only as intellectual property protection but also to generate quick return of investments (Table 2). Today, publishing results of publicly funded breeding research is often delayed until after patents have been secured.

### Corporate-based breeding

The corporate-based orientation is a style of thought that combines subjectivism and reductionism (see Fig. 4 and Table 1). It assumes that the truth and knowledge on what is best are relative and are up to the individual or corporate organization (i.e., “egocentric” view according to Bawden 2010). Similar to trait-based breeding, corporate-based breeding assumes that complexity can best be understood and dealt with by analyzing and breaking down complex crop characteristics into underlying single, manageable components and by specializing in those components that serve the company’s interests best. In that sense, the corporate-based breeding orientation often makes use of trait-based approaches. Corporate-based breeding is primarily focused on the continuity of the business and has to cope with competition, resulting in market-driven firms, which concentrate on those crops or globally adapted cultivars that provide the best profit. Corporate breeding companies may range from very small companies operating at national level to multinational companies operating at international level (Barnes et al. 2016). Essential in this orientation is the goal-driven, linear-economy thinking, including a top-down organization of the breeding and seed production activities.

There is an increasing need for a quick return on investments, making it necessary to protect the intellectual properties with patents on (parts of) the technology developed as is common in other industries (Howard 2015). It also leads to the application of breeding methods that create “natural” (biological) barriers to prevent the competitor to easily copy or multiply, such as is realized by incorporating the (cytoplasmic) male sterility trait in F1-hybrids. It also drives the policy to adopt seed regulations such as UPOV 1991 where farmers’ rights for on-farm seed saving are strongly restricted compared to the farmer-friendly former version UPOV 1978 (Braunschweig et al. 2014). There are also concerns about the mergers of the seed industry with the chemical industry resulting in forcing combined use of specific cultivars and crop protectants (Howard 2009).

The economies of scale are important drivers leading to a reduction of diversity in crop portfolio, mergers or consolidations to acquire economic vitality (Lindner 2004; Howard 2009). The research and development investments in corporate breeding companies are high (up to 35% of the yearly turnover) and the willingness of sharing knowledge with other players in the breeding sector is strongly reduced, which results in a lack of transparency (Barnes et al. 2016). The costs of investment increase as the lifespan of cultivars on the market becomes shorter. As diversity is key to food security and sovereignty, there is a need for green policies to allow diverse and local farmer participatory breeding programs to address small crops neglected in breeding (Khoury et al. 2014; Khoury and Jarvis 2014).

The strengths of corporate-based breeding include its entrepreneurship and innovative competiveness, and its emphasis on the market and value chain (Table 2). Simultaneously, these strengths also induce a narrow-mindedness and path dependency that should be classified as clear weaknesses. The increased focus of the corporate-based breeding orientation on the economic and legal value of seed as a commodity increases the tension with the drive in the community-based breeding orientation for developing open-source seed system models acknowledging the cultural value of seed including common good aspects, as described by Wirz et al. (2017).

### The need to combine potentials of the four paradigmatic orientations

Given the need to create ecological and societal resilience, these four breeding orientations each have their strengths and weaknesses (Table 2). They also have different values leading to different choices and decisions on trade-offs and risks (Table 1), none being inherently good or bad. Each understands, interprets, and operationalizes the six international breeding targets discussed in the introduction (agro-biodiversity; ecosystem services; climate robustness; food security, safety and quality; food and seed sovereignty; and social justice) in different ways. However, none of them alone can fully and whole-heartedly contribute to the realization of a productive, truly sustainable agriculture combining all those six targets. In reality, many, if not all, breeding organizations, companies, institutes, and societal initiatives integrate, often tacitly, various aspects of two or more quadrants (see also the interactions shown in Fig. 3). Some initiatives have been aiming to integrate purposefully several aspects of two or more quadrants (for example, Ceccarelli et al. 2001; Sperling et al. 2001). An interesting recent example that combines strengths of various quadrants is a case in China where F1-hybrids of maize were developed in a participatory program for the farmer fields in the more fertile valleys next to open-pollinated varieties for their rain-fed mountain fields; the institute breeders gave the farmers access to the parent lines and taught involved farmers how to propagate the F1-hybrid seed for sales to their local farmers’ market. In this way, F1-hybrids can serve smallholders while care is taken to secure their seed sovereignty (Li et al. 2013). Another example is the Dutch Bioimpuls program for the collaborative development of phytophthora resistant potato varieties where organic farmer breeders, commercial breeders and scientists work together. The scientists develop new germplasm to be further tested and selected by both farmer breeders and commercial breeders. Interesting selections by farmer breeders can be put on the market by the commercial breeders (Lammerts van Bueren et al. 2014).

However, these initiatives are not integrating the six sustainability targets to such an extent that all six can be substantially met. Also, these initiatives often meet various institutional hurdles preventing them to become part of mainstream breeding. And importantly, the relative size of these initiatives is limited compared to the size of the commercial breeding sector that is mainly situated in the lower two quadrants (corporate-based and trait-based breeding). In other words, a balance is needed between the efforts in the four quadrants.

To overcome the gaps that exist in the current breeding sector as a whole, it is necessary to capitalize on the emergent benefits of the positive interactions between the diversity of breeding models, breeding technologies and business models operating in a diversity of biophysical environments and at the same time dealing with a diversity in the societal aspects of our food systems. But it is also necessary to do more than combining the strengths and valuable assets of the different paradigmatic positions. To fulfill not only short-term needs of an increasing world population but also long-term societal and ecological resilience, integration of all four breeding orientations is urgently required, and this will not happen by itself. The commitment of cooperatives, institutions, society, and governments towards or the tendency to feel responsible for the long-term sustainability of society and agroecosystems is low, and thus, there is no urge to pay attention to sustainability measures that might increase costs on the short term but can ensure long-term sustainability of the planet and society, unless governments set boundaries and enforce new rules for creating a level playing field for all actors.

To meet the ambitious goals to ensure societal and ecological resilience at the same time, a more explicit type of thinking is required in the form of an overarching, integrative, fifth style of thought: systems-based breeding. This systems-based breeding orientation should provide a common future vision as a guide maintaining a balance between the four quadrants and, consequently, for a diversity of breeding approaches and activities that complement each other rather than a blueprint for one scheme. The systems-based breeding orientation should maximize the synergy between the strengths of community-based, ecosystem-based, trait-based and corporate-based breeding approaches, and develop innovative ways of organizing plant breeding and establishing stronger interactions between all components of the system to meet the future challenges as described in the introduction. This will be elaborated in the next section.

## Defining a systems-based breeding orientation

In the envisioned concept of systems-based breeding, “system” is defined as the space that encompasses the civil society (with its diversity of cultural norms and values), policy (with various governance institutions), nature (including the diversity of pedo-climatic conditions and habitats), agriculture (including the diversity of agro-ecosystems and farming systems), and value chains and markets as interrelated and mutually dependent components of the entire system (as shown in Fig. 3). Systems-based breeding aims to integrate various paradigmatic positions, but not to merge them. Systems-based breeding should be a style of thought that is systems-centric, both by its focus on holistic policy targets as well as by its methodology integrating holistic and reductionist approaches. The paradigm shift requires systems thinking of all actors involved, acknowledging that all parts and players in the system are interrelated and are affecting each other, and that all players need to commit themselves to a collective learning process to achieve this shift over the course of time (Senge 2006).

**Fig. 5:**
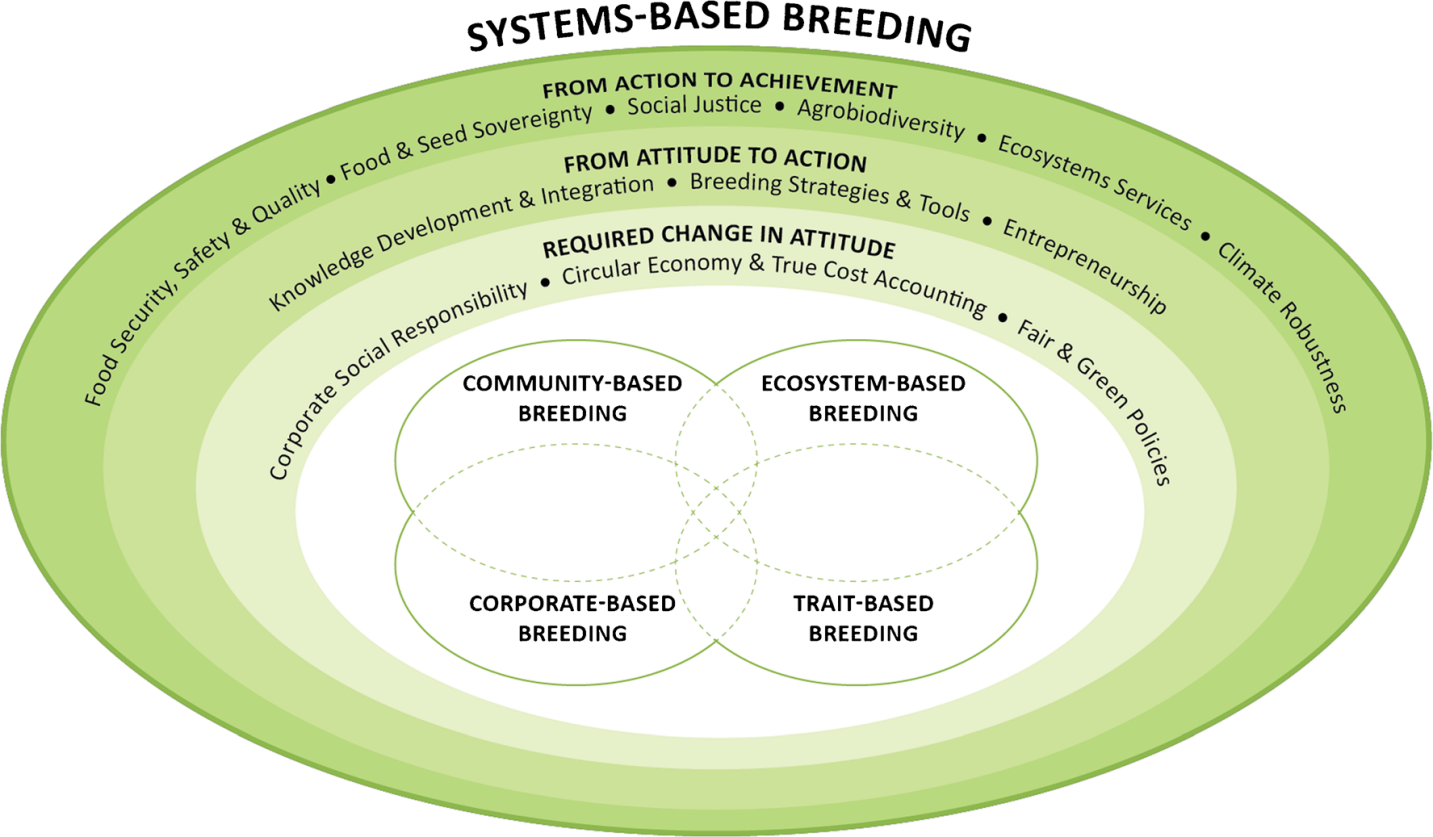
Representation of systems-based breeding as a fifth, overarching breeding orientation integrating the strengths of the four breeding orientations earlier described

To define the systems-based breeding orientation, we will identify and discuss key aspects of required change in attitude (corporate social responsibility, circular economy and true-cost accounting, fair and green policies), the process from attitude to action (knowledge development and integration, breeding strategies and tools, entrepreneurship), and the process from action to achievement (food security, safety and quality; food and seed sovereignty; social justice; agrobiodiversity; ecosystem services; and climate robustness). These three areas will be discussed below and are summarized in Table 3 and Fig. 5.

**Table 3:** Key elements and aims of the systems-based breeding orientation

	Key elements	Aims
Required change in attitude	Corporate social responsibility	Including ethical and social responsibilities beyond legal and economic responsibilities
	Circular economy and true-cost accounting	Rearranging linear relationships such that value chains become value networks in which various actors work together
	Fair and green policies	Creating a frame work for optimal integration of all components of systems-based breeding
From attitude to action	Knowledge development and integration	Supporting continuous development of specialized, generalized and integrated knowledge at various levels (socio-economic, agro-ecological, etc.)
	Breeding strategies and tools	Designing a range of different appropriate technical breeding approaches
	Entrepreneurship	Developing sound entrepreneurial models suitable for various small and large value chains
From action to achievement	Food security, safety, and quality	Enhancing breeding of food that is healthy, nutritious and safe, with high and stable yield, and good shelf-life that does not require chemicals during production and storage
	Food and seed sovereignty	Allowing a pluriformity of breeding models to co-exist and for communities and markets to choose breeding models that fit best, implicitly serving cultural diversity and seeds as common good
	Social justice	Fair and just assigned rights and duties in relation to breeding activities and products, such as breeders’ privilege, farmers’ rights and fair prices for (farmer) contract seed producers
	Agrobiodiversity	Enhancing agro-biodiversity in farming systems; within and among crop species; improve diversity in major and small crops
	Ecosystem services	Improving breeding strategies, breeding products and crop traits that support ecosystem services
	Climate robustness	Creating climate robust and flexible breeding strategies and products that provide yield and quality stability under variable conditions

### Required change in attitude

To allow all players to adopt systems thinking, there is a change in attitude required, not only from the private businesses but also from citizens and policy makers. There are already starting points to build on, such as corporate social responsibility, circular economy and true-cost accounting, and fair and green policies, but efforts will need to be intensified in the context of the systems-based breeding orientation.

Corporate social responsibility (CSR) is a form of corporate self-regulation, integrated into a business model, monitoring and ensuring its active compliance with the spirit of the law, ethical standards and national or international norms (Knowles 2014). It requires ethical, philanthropic, legal, and economic responsibilities, but also leadership, personal responsibility, and trust (Mostovicz et al. 2011). CSR within a breeding company could entail balancing people, planet, and profit, respecting diversity of societal norms and values, while interacting with society to enhance choice and appreciation of breeding approaches and produce supporting sustainability.

An important aspect of sustainability is the circular economy that should go beyond a regenerative system in which resource input and waste, emission, and energy leakage are minimized by slowing, closing, and narrowing material and energy loops as the social feedback loops are missing (Geißdörfer et al. 2017). Van der Weijden et al. (2012) considered it a precondition for an integrated sustainable food production system to move away from the linear organized production chain based on specializations where crucial relationships have gone lost or have become corrupted. Only when there is an overall agreement in the chain of the common goals of sustainability, externalities will be prevented; so rebuilding relationships in the value chain into food networks, and interweaving ecology, society, sustainable agriculture and healthy food are key. In such new relationships, true-cost accounting can contribute to enhance transparency about hidden costs by showing the ecological and social (health and justice) costs made and the benefits realized during production, storage, processing, transport, marketing, etc. This is now being implemented for food products by several food organizations (see, e.g., Holden 2013; Eosta et al. 2017).

The term “fair and green policies” underlines that sustainable food production and consumption need to be embedded and enhanced by good governance and policies to create a level playing field for all players and a legal and economic framework for long-term green and fair innovation. For example, most companies are specialized in a specific assortment of the main crops and value chains. Government policies should stimulate a diversity in initiatives, not only to stimulate breeding for small crops and markets (Khoury and Jarvis 2014), but also to stimulate different breeding approaches for the main crops. An example is enhancing sustainability by making use of genetic diversity, such as multiline breeding (Groenewegen 1977; Mundt 2002) or evolutionary breeding, as is currently applied in the organic sector with composite cross populations (Döring et al. 2011). This requires adaptation of the variety registration based on the so-called DUS (distinctness, uniformity, and stability) protocols accepting heterogeneous material instead of only departing from homogeneous pure lines (Louwaars 2018).

### From attitude to action

Capra (1997) suggested that people need to understand the principles of the organization of ecosystems and need to become “ecoliterate” to create sustainable human communities. To achieve that, shared and reflective learning is needed to reconnect people and food (King 2008). King and Powell (2000) indicated that ecological and societal resilience encompasses plasticity, also in non-equilibrium systems, but also the capacity to co-evolve and co-learn, managing cyclical patterns and non-linear processes with multi-stakeholder teams.

Plant breeding and seed production can only successfully contribute to the systems-based breeding targets (see Section 4.3) when this vision of a coherent and system-centric breeding concept is shared and nurtured. This process not only requires respect for the pluriformity in society but also empowerment of diversity of approaches to reach ecological and societal resilience. It requires a diversity of actions from various groups in a pluriform society: (i) knowledge development and integration, (ii) development of a multitude of breeding strategies and tools, and (ii) entrepreneurship. The first action concerns continuous knowledge development and sharing on all aspects of systems-based breeding, and creating synergy of social and natural sciences necessary to combine sustainability, justice and resilience**.** A proper integration of specialist knowledge, generalist knowledge, technological choices, and socio-economic and cultural aspects will be crucial. This will require a joint learning process based on transdisciplinarity and action research methods ultimately leading to breeding strategies and tools that contribute to socio-technical innovations at various levels: society, value chain, agro-ecosystem, farm, and trait. Systems-based breeding should be an integrated part of systems innovations in agriculture. Breeding strategies for diversification in agriculture can combine mainstream approaches with those emerging in various research experiments, such as multi-lines (e.g., Henry et al. 2010; Lynch 2011), evolutionary breeding (e.g., Döring et al. 2011; Murphy et al. 2016; Raggi et al. 2017) and breeding for mixed cropping systems (Davis 1989). But many knowledge gaps still exist in implementing such methods in commercial breeding programs, and practical farming and processing practices, and in a legal framework that, for example, allows heterogeneous material to be marketed.

Ideas addressing social justice within the seed business, such as paying contract farmers producing seeds for the corporate companies a fair price, or models that share corporate profit among employees should be further developed. Also, the current initiatives on seed sovereignty in breeding models need to be further explored to identify ways to finance breeding activities other than through royalties of seed sales (Osman et al. 2007; Kloppenburg 2010; Wirz et al. 2017). This requires novel entrepreneurships including all the skills required to develop sound entrepreneurial models in breeding and seed production suitable to diverse small and large value chains and markets (FAO 2016).

### From action to achievement

The aim of systems-based breeding is ecological and societal resilience by combining food security, safety and quality, food and seed sovereignty, and social justice without jeopardizing long-term sustainability of the ecological context by enhancing agrobiodiversity, ecosystem services, and climate robustness. Many of these targets are interrelated. For instance, social justice cannot be realized without environmental sustainability (Coote 2014).

We describe these six targets in terms of breeding goals (see Table 3). For food security, safety, and quality, plant breeding should develop cultivars and populations with high, stable yields of safe, nutritious, diverse food with good storability and shelf-life that do not depend on chemical treatments for good performance. Food and seed sovereignty also means communities should be able to define their own means of production or acquisition and their own preferred food and seed, considering food as a basic human right and seeds as part of common goods (De Schutter 2014; Wirz et al. 2017). De Schutter (2014) also pleads for more support for participatory plant breeding in various crops and regions to meet such goals. Social justice is an important target to contribute to societal resilience and includes the need for plant breeding and seed production to be organized in such a way that fair distribution of wealth, and equal opportunity among communities can be ensured.

To contribute to the long-term ecological resilience in our food systems, agrobiodiversity and ecosystem services should also be addressed by plant breeding. Agrobiodiversity includes not only diversity of crop species but also genetic diversity within crops, to support sustainable and nutritious food production systems and diversity of markets and cultures. It also includes investments in breeding of small crops (Khoury and Jarvis 2014). Ceccarelli (2015) and Coomes et al. (2015) argued that involving various actors in the breeding process supports agrobiodiversity. Ecosystem services regulate and support the environment within agro-ecosystems and sustain future food production and therefore should play a role in agronomy and plant breeding (see, e.g., Huang et al. 2015). Recently, the FAO pictured how livestock species and breeds could contribute to ecosystem services and biodiversity (Hoffmann et al. 2014) but even plant breeders have not internalized ecosystem services yet to a large extent (see Section 3.2). Some of these characteristics related to ecosystem services, such as plasticity of root systems for resource capture, will contribute also to climate mitigation and climate robustness (e.g., Kadam et al. 2017). Climate robustness requires adaptation to variable, unpredictable, and extreme weather conditions tailor-made to local contexts (Shi et al. 2017). The main characteristics of climate robustness are yield stability and plant phenotypic plasticity (Ceccarelli et al. 2010; Nicotra et al. 2010).

## Discussion and conclusions

In this paper, we have made a plea for an integrated approach, which we have coined the systems-based breeding orientation; we propose it as a framework where different approaches related to the four quadrants of Fig. 4 can be integrated. A first outline for such a methodological framework, allowing the integration of different approaches, such as, for example, positivism and post-modernism, has been described by Nuijten et al. (2013).

Very recent developments in agriculture have already sown the seeds of this systems-based breeding approach aiming at productivity based on healthy economic performance, transgenerational sustainability and justice, and ecological and societal resilience. Under pressure of consumer organizations, farmers and breeders alike are forced to demonstrate a societally relevant *Purpose* in addition to serving the needs and concerns of *People*, *Planet*, *Profit* in order to keep their license to operate (Geißdörfer et al. 2017). We have demonstrated that the plant-breeding sector needs to make progress to contribute significantly to *Purpose* and all its aspects of sustainability, through diverse and complex socio-technical transitions (Geels and Schot 2007). In this section, we will reflect on some critical issues in our concept that were raised in discussions with breeders.

### Diversity of breeding initiatives and variety registration protocols

Moving towards systems-based breeding requires multiple processes of co-learning and co-constructing and a diversity of breeding initiatives. Initiatives may start with those willing and eager to become frontrunners to realize examples that create experience on how to overcome obstacles. Initiatives may start from within existing breeding organizations or companies and new initiatives can also start at niche level. Both are important, have different roles and should interact. Initiatives from within the existing breeding sector may have more impact by their size, whereas initiatives starting at niche level can help make significant forward-looking changes. To foster change, fair and green policymaking is crucial. The interplay of the existing sector, the niche and policymaking is important to make a transition to systems-based breeding feasible (see Geels 2011).

King (2008) argued that enhancing ecological and societal resilience requires more than current alternative systems (such as organic agriculture, permaculture, etc.) can offer. None is the “best,” as there is a need for a diversity of agro-ecological systems that together bring about the variation of functions at multiple scales that is required to build true resilience. Gunderson and Pritchard (2002) argued that no single mechanism can guarantee maintenance of resilience; they expressed the opinion that in ecological systems resilience lies in the requisite diversity of functional groups and accumulated ecological capital that provides sources for recovery. This certainly applies to plant breeding. Depending on the ecological and cultural context different choices will be made, and trade-offs will be weighed differently. In plant breeding, agrobiodiversity also implies a diversity in breeding approaches and in seed systems (Louwaars et al. 2011; Louwaars 2018).

In addition, institutional change is required to give space to a multitude of innovations. Typical examples are the variety registration protocols. They are based on the so-called DUS principles: distinctness, uniformity, and stability and on the Value for Cultivating and Use (VCU) testing under conventional growing conditions. These protocols impede innovation in the systems-based breeding orientation and should therefore be reconsidered. Already several European countries have adopted the protocols for testing and registration of cereal cultivars that are adapted to organic or low-input conditions, by including important features, such as weed suppression ability and disease resistance (Osman et al. 2015).

### Seeds as common good

Other important aspects of institutional arrangements associated with genetic diversity and food sovereignty are intellectual property rights on plants (IPR). From a systems-based breeding orientation, free access to genetic resources is considered important, as it provides the best conditions for a broad genetic base to enhance food security and food sovereignty. However, the way plant varieties have been developed, released and distributed has changed drastically over time: from publicly freely available resources created by state institutions to highly protected material, produced and managed by the private breeding sector. Since the 1970s, governments have largely withdrawn from public breeding activities and left breeding in the hands of the private sector. Breeding for resistance or tolerance to biotic and abiotic stresses is complex and costly, and hence is currently considered risky. Therefore, the current private business models in breeding require greater financial incentives based on more restrictive IPR to enable these companies to invest in broadening their genetic base (Donnenwirth et al. 2004). A group of non-governmental organizations published a report entitled “Owning seeds, accessing food,” in which they expressed their concern that stronger plant variety protection as agreed upon in UPOV 1991 (the 1991 declaration of the International Union for the Protection of New Varieties of Plants (UPOV)) will threaten the right to food for smallholder farmers. They therefore plea for a thorough review of the UPOV rules, and especially for a revision of those aspects of the UPOV rules that impede the informal seed sector and damage the interests of (farmers in) low-income countries (Braunschweig et al. 2014) and high-income countries (Kloppenburg 2010; Luby et al. 2015).

In response to such developments, many new initiatives arose. For example, the Open Source Seed Initiative (OSSI) in the USA explores alternative strategies by promoting and maintaining open access to plant genetic resources worldwide and increases the common pool of plant genetic resources (Kloppenburg 2010; Luby et al. 2015). However, such approaches do not yet solve the issue who is going to pay for the breeding efforts and requires a broadened view.

Wirz et al. (2017) distinguished three types of goods for seed and plant varieties: cultural, economic, and common good. These authors made a plea to create a balance between these three goods. They also stressed that seed is different from other threatened natural resources, as it gets lost when it is not used. In fact, seed is not only a natural resource, but also a cultural and economic resource. They also call for designing a better balance between public and private breeding activities to allow more diversity in regional-based breeding activities including conservation of traditional crops and varieties.

There are many other examples emerging where partners in the value chain experiment with ways to become co-creators of genetic diversity and share the responsibilities for developing and maintaining new varieties rather than merely acting as donors (see, e.g., Osman et al. 2007; Kotschi and Wirz 2015). This requires new relationships within the value chain including breeders as partners in the food chain or food networks. Fair and green policies should support the exploration of such new breeding organizations or networks. Also, policy support is needed for speeding up the emergence of start-ups which set up new breeding programs to close the gap for small crops and small markets to safeguard diversity in the fields and in our diets.

### New entrepreneurial models

Carroll and Shabana (2010) described a possible transition from a narrow view on business strategies focusing on immediate cost savings to enabling a firm to enhance its competitive advantage and create win-win relationships with its community of stakeholders, while at the same time realizing gains from cost and risk reduction and legitimacy and reputation benefits. However, including corporate social responsibility (CSR) not automatically leads to sustainability (Knowles 2014). CSR looks backwards at performance, typically over the last 12 months, while sustainability is forward-looking.

There are examples of companies that want to show top leadership in transitioning towards a more integral sustainability plan (Grayson 2011; Unilever 2017). A Dutch company trading in organic fruits, called Eosta, recently introduced true-cost accounting into its entrepreneurial model (cf. Holden 2013; Eosta et al. 2017). Such integral sustainability thinking should be extended to breeding companies with respect to prioritizing long-term breeding goals.

Wolfe et al. (2008) described various types of breeding programs for organic agriculture ranging from combining conventional and organic markets to fully committed to the organic market. However, producing organic seeds of an existing variety assortment is one thing, but remodeling a breeding program so that it produces varieties suitable for more sustainable farming systems, such as organic agriculture, is another and more radical issue, involving different breeding priorities and selecting under different crop management conditions (Osman et al. 2016; Kokare et al. 2017). Changing breeding priorities also brings up the question to what extent certain cultivar traits are in the interest of a seed company that wants to sell seeds every year. It is a commonly shared secret that potato-breeding companies consider it not in their business interest to select for a high level of virus resistance in potato as a certain level of susceptibility prevents farmers from saving their own seed too often (pers. communication P. Keijzer, 2018). Similarly, persistency of perennial fodder crops like red clover is at the moment not a priority for seed companies (Hoekstra et al. 2018), while persistent cultivars would positively influence soil organic matter build-up, carbon sequestration and soil biodiversity (Van Eekeren et al. 2008). Such dilemmas touch upon the radical change needed in corporate social responsibility when developing a systems-based breeding orientation.

### Shared sense of urgency

In order to trigger fundamental change in the global food systems and in the role of plant breeding in those systems, it is necessary that there is a widely shared sense of urgency for change, also in the objectives of plant breeding. Rockström et al. (2009) indicated that nine planetary boundaries were already overstepped and stressed the urgency to mitigate at least three out of those nine planetary boundaries. These three included biodiversity loss (at species level), climate change, and human interference with the nitrogen cycle. These three overstepped boundaries all have a very close relationship to agriculture. These authors argued that these rates of change cannot continue without significantly eroding the resilience of major components of the earth-system functioning. Especially climate change may hurt agriculture tremendously. The resources to be used to cope with such threats are also becoming increasingly scarce, such as diversity of crops (Khoury and Jarvis 2014) and of the crop wild relatives necessary for breeding (Castañeda-Álvarez et al. 2016).

Urgent innovations can be catalyzed by innovation brokers (Klerkx and Leeuwis 2009). However, the success of innovations also depends on historical and socio-economic contexts (Geels and Schot 2007). Nuijten et al. (2017b) analyzed various cases of introducing in different ways disease resistant cultivars of apple and potato into the market; such introductions were considered urgent in order to enable sustainable organic production of such produce. Nuijten et al. (2017b) formulated key lessons learned from the cases: (i) there must be an urgent need creating a pull strength; (ii) for creating enough pull strength, it is important to involve several stakeholders; (iii) involving all stakeholders requires a shared language and a common culture; this can help to also create push strength; (iv) without adequate push strength, no progress will be realized.

Systems-based breeding goes a step further and also takes into account long-term societal and ecological benefits that go beyond the direct interest of the value chain. The latter also requires a sense of urgency among the policy makers and the general public, to realize fair and green policies that support appropriate, sustainable innovations.

### Perspective

The systems-based plant breeding orientation as defined in this paper will allow plant breeders to not only catch up but also become initiators for more cooperation in making agriculture more ecologically and societally resilient.
